# Urban growth’s implications on land surface temperature in a medium-sized European city based on LCZ classification

**DOI:** 10.1038/s41598-024-58501-0

**Published:** 2024-04-09

**Authors:** Aleksandra Zwolska, Marek Półrolniczak, Leszek Kolendowicz

**Affiliations:** Department of Meteorology and Climatology, Adam Mickiewicz University in Poznań (Poland), Krygowskiego Str., 10, 61-680 Poznań, Poland

**Keywords:** Climate sciences, Climate change, Environmental impact, Sustainability

## Abstract

The study determined the influence of changes in land use and land cover (LULC) on land surface temperature (LST) over a 33-year period based on a medium-sized European city (Poznań, Poland). The LST was estimated from Landsat 5, 8 and Terra (MOD11A2v6) satellites. The local estimation of climate patterns was based on the Local Climate Zones (LCZ) classification utilised with the methodology proposed by the World Urban Database and Access Portal Tools (WUDAPT). Moreover, the Copernicus’ imperviousness density product (IMD) was used. Between 2006 and 2018 the area with IMD of 41–100% increased by 6.95 km^2^, 0–20% decreased by 7.03 km^2^. The contribution of built-up LCZs increased by 7.4% (19.21 km^2^) between 1988 and 2021 reaching 13% (34 km^2^) within open mid-rise LCZ. Due to urbanisation and reforestation, low plants LCZ shrunk by 12.7%. For every 10% increase in IMD, LST increases by up to 0.14 °C. Between 1988 and 2021 the LSTm in specific LCZs rose from 1.52 up to 2.97 °C. As per LST models LCZ change from natural to built-up led up to 1.19 °C LST rise. The increase of the LSTm was registered even when the LCZ remained unchanged.

## Introduction

Climate change has been shown to elevate urban temperature, intensifying extreme heat events, increasing their frequency, duration and strength, and raising the risk of heat exposure in the cities^1,2^ negatively influencing ageing societies' health and urban environment^3–5^. It is also the main ecological driver of the hydrological balance^6,7^. In urban areas, temperatures can reach even a dozen degree Celsius higher than in rural^2,8,9^. With a constant projected increase of the extreme heat events’ intensity/frequency, urbanisation and urban heat island (UHI) influences the mortality along with economic losses^9,10^. Progressing urbanisation creates a need to better understand the impact of cities’ fabric on the urban interior environment and urban planning strategies in mitigating the increase of both air and surface temperature (LST), e.g. by including new green and water areas in the city^11,12^. According to Yang et al.^13^ the balance between urban expansion and thermal environment quality is the key to ensure sustainable urban development. Since the medium-sized cities (< 1 million urban population) are the habitat of over the half of the world’s population and are facing the risk of experiencing extreme urban heating effects, the urban climate research should be focused on them and continued^14^.

It is known that the impervious surfaces increase the risk of the floods in urban areas due to insufficient drainage^7^ and that the spatial arrangement and structure of the impervious surfaces (their shape and size) are having several impacts on LST. The higher the spatial density of the impervious areas the higher the increase of LST compared to natural areas^15–17^. To describe its patterns and impacts in a more detailed way there is a need to take into consideration the impact of specific land use and land cover (LULC) forms on LST. Previously the terms “urban” and “rural” were used to describe urban climate^18,19^. The aforementioned terms raised several problems and compared the differences in LST/UHI patterns rather generally since there were no universal definitions of them^20^.

The constant urban development and its impact on people living in the cities created the need to work on a method to standardise the research on urban climate. Stewart and Oke^21^ developed the Local Climate Zones (LCZ) classification. Local climate of the city, as defined by the local climate zones classification, is the unique expression of climate patterns influenced by specific LULC characteristics. The classification consists of 17 typological units based on properties of surface structure (height and density of the structures) and surface cover (including its perviousness and imperviousness). The following are taken into account: sky view factor, aspect ratio, building surface fraction, impervious surface factor, pervious surface fraction, the geometric mean height of roughness elements, and terrain roughness class^21^. The LCZ classification is widely used in studies on urban climate; in estimating a correlation between the urban morphology and LST and UHI^22–25^, to detail studies on heat and cold waves in urban areas^26,27^, in the estimation of the impact of LCZ changes on LST^28,29^ as well as in the works focused on the methodology of the classifying urban areas^30–34^. The World Urban Database and Access Portal Tools (WUDAPT), developed the procedure, and later the online generator, to carry on LCZ classification in a simple and objective way^30,31,35^. The WUDAPT procedure used in this study, allows the classification of the urban areas and the determination of the spatial range of distinguished LCZ classes based on commonly available and free Landsat data. For the estimation of the thermal differentiation of distinguished LCZs Landsat 5 and Landsat 8 data are used^24,36^. According to Lehnert et al.^37^ remote sensing LST measurements are used for the thermal analysis of LCZ in 17% of the studies. Besides the satellite imagery, other researchers also use in-situ data^26,38^ or both^35^.

The most popular sensor used for the research on LST is Moderate Resolution Imaging Spectroradiometer (MODIS)^39^. The entire Earth surface is orbited every one to two days utilising 36 bands with spatial resolution from 250 to 1000 m^40^. However, in the research on the anthroposphere Landsat satellites take over. The Landsat mission includes eight generations of satellites. Landsat products are characterized by higher spatial resolutions, from 30 to 120 m, but lower temporal resolution, with revisits every ~ 16 days. However, Landsat starts from 1982 and provides the longer data series than MODIS^39,40^. Studies on urban areas are focused on characterising the difference between urban and non-urban areas which does not require high temporal resolution^39^ but higher spatial resolution. In this study MODIS 8-day LST product was used in modelling coupled with the Copernicus imperviousness density product (IMD). The IMD product was used also by Demuzere et al.^41^ in mapping of LCZs for Europe. In our research it allowed us to estimate the influence of IMD on LST and hence the potential LST change due to transformations from one LCZ to another.

**Figure 1:**
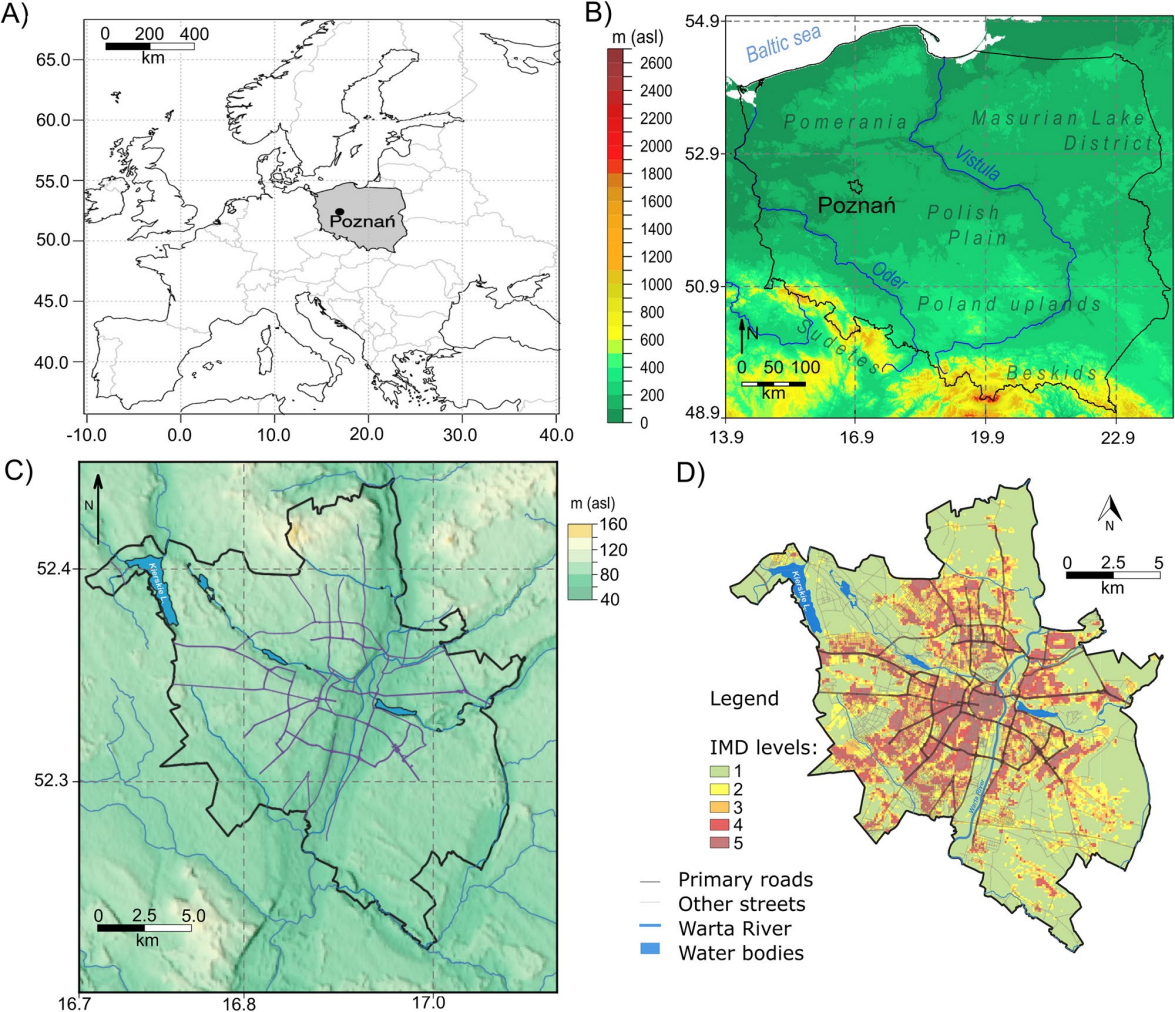
The research area: localization of Poznań (**A**,**B**), elevation map of Poznań (**C**) based on Shuttle Radar Topography Mission (SRTM) (90 m)^45^ (https://srtm.csi.cgiar.org/) and the spatial distribution of IMD levels in Poznań in 2018 on the base of Copernicus’ Imperviousness Density (IMD) product (IMD levels: level 1 IMD 0–20%, level 2 IMD 21–40%, level 3 IMD 41–60%, level 4 IMD 61–80%, level 5 IMD 81–100% (**D**).

Poznań (52° 18′ N–52° 30′ N, 16° 48′ E–17° 04′ E) is eighth in terms of area (261.8 km^2^), and fifth in terms of population in cities in Poland (541 316, stated at 31.12.2022)^42^. The city is located in Central Europe on an elevation between 60 and 154 m asl (Fig. 1). Poznań is situated in a temperate climate zone, transitional between oceanic and continental. The annual average air temperature in Poznań is 9.5 °C (1991–2020), with the highest temperature in July (19.5 °C) and the lowest in January (− 0.4 °C). The average annual precipitation is 539 mm, with the highest in July (84.4 mm) and the lowest in February (30.7 mm). Due to the location, the most common advection brings the polar air masses from the west (around 70%); polar continental, arctic, and tropical air masses are significantly less frequent^43,44^.

The studies carried out so far in Poznań confirmed occurrence of the UHI phenomenon that was investigated based on in situ measurements and remote sensing data (SUHI). Remote sensing data were used also in air temperature modelling. An increased frequency and intensity of heat waves as well as a decrease in the number of cold days and cold waves was noted^44,46,47^. The research of Półrolniczak et al.^48^ found the intensity of UHI significantly higher in anticyclonic conditions. In preliminary research using Corine Land Cover (CLC) database^49^ it was also confirmed that anthropogenic surfaces in Poznań increased at the expense of agricultural areas from 46.8% in 1990 to 57.5% in 2018.

Each city is unique, has a distinct environment, geography, structure and its own specific local climate. Accordingly, it is still important to develop the climate research in the cities, especially considering the uncertainties of the directions of urbanisation in the future^50^. Our research, examining shifts from the past to present, aims to enhance our understanding of the relationship between urban structures and local climate. We prioritised capturing the influence of the urban growth, hence in the research instead of 30 years of climatological cycle we utilised the data based on the maximum temporal availability to estimate the LULC changes. The selection was also limited by the requirements like similar weather conditions and lack of cloud cover over the research area, thus the utilised data does not coincide with the beginning of the Landsat 5 activity. Taking into account LCZ classification and remote sensing data we can address the goal of the research in assessing how changes in LULC over 33 years influenced LST in the medium-sized Central European city. Until now similar research in Europe has not been conducted. Poznań belongs to the group of the medium-sized expanding cities, which is the most vulnerable to the risk of experiencing extreme urban heating effects^14^. In the last 30 years, the annual mean air temperature trend in Poznań has shown an increase of + 4.6 °C per 100 years^43^. Our research will help to understand the urban environment better and draw conclusions for spatial planning strategies as well as urbanisation law regulations, and improving the citizens’ well-being^12^.

## Methods

**Figure 2:**
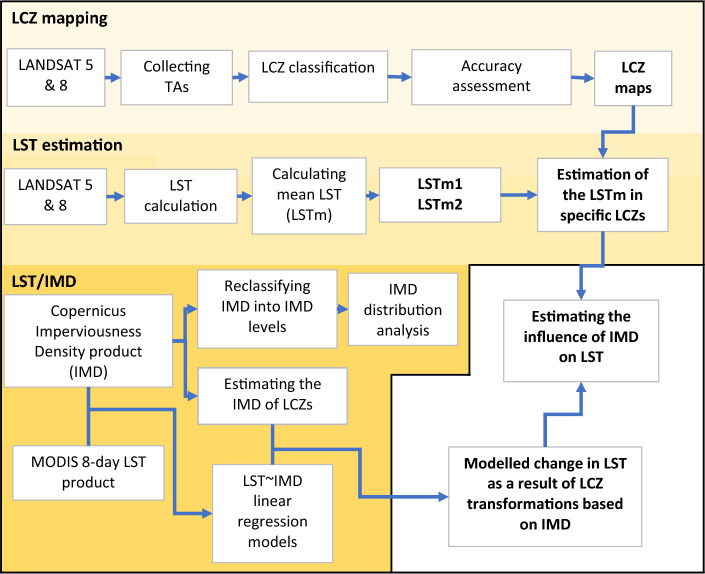
Flowchart illustrating the sequential stages of data collection and analysis in the study.

Figure 2 provides a graphical representation of the procedural framework used in our research. It consists of the key steps used in estimation of the urban growth’s implications on LST.

### Relationship of land surface temperature and the imperviousness of the surface

The study used the MOD11A2v6 product (in 1 km spatial resolution) from the Terra MODIS collection and Copernicus' IMD product (https://land.copernicus.eu) (in 100 m spatial resolution), to explore how impervious surfaces affect LST. The MOD11A2v6 provides an average 8-day LST and Emissivity. The product is generated from daily MOD11A1 data with temporal extent 18.02.2000 to 16.11.2022^40^. The 3-yearly IMD product contains data about the imperviousness density over urban areas and is generated using a semi-automatic classification based on the Normalized Difference Vegetation Index (NDVI). The IMD is available for the reference years 2006, 2009, 2012, 2015 and 2018 in the spatial resolution of 10 m (2018), 20 m (2006–2015) and as aggregated of the aforementioned—100 m products^51^. The IMD product with the 100 m resolution was successfully used in research on mapping of local climates of urban areas along with the LCZ classification by Demuzere et al.^41^.

The daytime LST MOD11A2v6 Terra MODIS data from January 2002 till December 2021 and IMD for 2006 (3-yearly product with temporal extent 2005–2007) and 2018 (3-yearly product with temporal extent 2017–2019) were acquired through the NASA server (https://urs.earthdata.nasa.gov/home) with the use of R programming language and its package MODIStsp^52,53^, and Copernicus database (https://land.copernicus.eu) respectively^51^. The MODIS data were converted from MODIS units to LST in degree Celsius by applying the MODIS scale factor^54^. The LST data were then divided into decades: 2002–2011 and 2012–2021 and resampled to the resolution of IMD data (100 m). The changes in the percentage of impervious areas between 2006 and 2018 were investigated along with the LST between the chosen decades. The relationship between IMD and LST was tested using the linear regression model. In order to facilitate the quantitative and spatial interpretation of IMD distribution changes, the values of IMD (0–100%) were reclassified into IMD levels: level 1 (IMD 0–20%), level 2 (IMD 21–40%), level 3 (IMD 41–60%), level 4 (IMD 61–80%), level 5 (IMD 81–100%) (Fig. 1B).

### LCZ procedure

To standardise the documentation of observations and to facilitate the comparison of results of the research LCZ classification was used^21^. Its globally unified LULC classes make the results of the research applicable for any urban area of the same climate zone. In order to determine LCZ classes for Poznań the WUDAPT L0 LCZ procedure was applied^30^. The procedure is carried out with the use of Google Earth and SAGA GIS and consists of three main operations: pre-processing of the satellite raster data, digitization of relevant training areas (TAs) and application of the classification’s algorithm in SAGA GIS^55^. To make both classifications (for 1988 and for 2021) comparable, both were generated on the base of the same TAs^29^.

To conduct LCZ classification with WUDAPT L0 procedure cloud-free satellite images corresponding to various seasons^55^ from Landsat 5 and Landsat 8 from the US Geological Survey^40^ were obtained for: 17 April 1988, 28 May 1988, 26 October 1988, 28 April 2021, 8 June 2021, 30 October 2021. The selected multi-temporal datasets were pre-processed: cropped to the regions of interest (ROI) boundaries and resampled to 100 m^55^.

The second input to the classification are TAs digitized according to the WUDAPT guidelines^56^. Collecting TAs from locations whose LULC category did not change eliminated the effect of differences in ground truth data on the accuracy of mapping^29^. On the research area, 8 LCZ categories according to LCZs were identified. Five of them represent land use-based categories: compact mid-rise (LCZ2), open mid-rise (LCZ5), open low-rise (LCZ6), large low-rise (LCZ8), and heavy industry (LCZ10) and three land cover-based LCZs types: dense trees (LCZA/LCZ101), low plants (LCZD/LCZ104), and water (LCZG/LCZ107). Another step was the application of the classification algorithm in SAGA GIS. The algorithm used in the WUDAPT procedure is a Random Forest (RF) classifier. It consists of decision trees designating every pixel of the image into LCZ type. It is referred to as RF because its subsets are created randomly, and the predicted value is the mode of prediction from all trees^29,55^. The RF requires a small amount of training data while still providing valuable results and also manages to process a large amount of data, without any removal. It is not sensitive to overtraining and is preferable for GIS data and multiple source remote sensing datasets^28,57,58^. The appearance of the final LCZ map in WUDAPT highly depends on the applied post-classification filter^55^. For Poznań, the optimum choice was to use a majority filter of the 3 pixel radius.

Accuracy assessment of LCZ is essential to separate the real changes from changes caused by errors. To generate indicators of accuracy at the class level confusion matrix was used while at the level of the entire study area it was Overall Accuracy (OA). We used 60% of the dataset as the training data and remaining 40% as the test data. The use of OA with respect to the ground data is the most common and valid way to assess accuracy^29,59^. Besides the aforementioned, accuracy was also assessed by overlying generated LCZ maps with Google Earth imagery.

The mean IMD (%) was estimated for each LCZ class of 2021. That allowed the estimation of the potential size of the IMD (%) change due to the transformation from one LCZ class to another. Next, based on these results and the result of created IMD ~ LST models, focusing on the influence of IMD change on LST, the predicted change in LST as a result of transformation from one LCZ class to another has been estimated.

### Landsat LST

To calculate the LST for established LCZs, the Landsat 5 and 8 images were used. Landsat was chosen because it offers data with high spatial resolution. Firstly, the pre-processing procedure was done by applying an atmospheric correction. It is a crucial step in preparing the satellite products to work with on LST retrieval^60^. Next, the brightness temperature and emissivity were calculated in QGIS^61^ along with applying DOS1 atmospheric correction on selected satellite images. The emissivity of the research area was estimated after calculating the fractional vegetation cover on the basis of NDVI^62,63^. The actual LST of the research area was then calculated after applying the emissivity correction on brightness temperature^64^. In order to calculate the mean LST (LSTm) of the research area both in the past and in the present several satellite images were selected: 5 for 1987–1989 (LSTm1) and 5 for 2020–2022 (LSTm2) (Table 1). Firstly, the LST for each satellite image was calculated then the LSTm1/LSTm2 was estimated as their mean. The LSTm1/LSTm2 were then resampled to the resolution of LCZ (100 m)—it was confirmed that the 100 m resolution LCZ information provides necessary information for urban planning and ecological city evaluation—not only at mesoscale but also local and microscale^65^. Satellites passing at comparable times over the study area provide images obtained from the same angle and with the similar instrument sensitivity. The LST calculations conducted with the same methodology guarantee similar accuracy of obtained LST for both periods. Moreover, the imagery was taken on corresponding dates and weather conditions: similar values of air temperature and humidity and their daily patterns, all-day wind velocity ≤ 6 m/s and no precipitation for at least 6 h before the satellite’s revisit. The satellite images were selected taking into consideration the hourly data from the meteorological station Poznań-Ławica belonging to the Institute of Meteorology and Water Management—National Research Institute. All the estimations were obtained using R programming language^53^.

**Table 1:** Landsat images used in LSTm1 and LSTm2 calculations.

Time period	Imagery	Date	Time	Path/row	Scene ID
1987–1989	Landsat 5	17/04/1988	09:20:19 UTC	191/23	LT51910231988108KIS00
	Landsat 5	15/05/1989	09:12:55 UTC	190/24	LT51900241989135FUI00
	Landsat 5	26/05/1987	09:08:30 UTC	190/24	LT51900241987146XXX02
	Landsat 5	19/08/1989	09:11:00 UTC	190/24	LT51900241989231FUI00
	Landsat 5	06/10/1989	09:10:00 UTC	190/24	LT51900241989279FUI00
2020–2022	Landsat 8	28/04/2021	09:49:46 UTC	191/23	LC81910232021118LGN00
	Landsat 8	10/05/2022	09:44:18 UTC	190/24	LC81900242022130LGN00
	Landsat 8	20/05/2020	09:43:50 UTC	190/24	LC81900242020141LGN00
	Landsat 8	08/08/2020	09:44:22 UTC	190/24	LC81900242020221LGN00
	Landsat 8	11/10/2020	09:44:44 UTC	190/24	LC81900242020285LGN00

In order to estimate the magnitude of differences amid individual LCZ units and to evaluate the correctness of the application of the LCZ classification, LST statistics and statistical tests were applied. The non-parametric Kruskal–Wallis test was used for all groups and the non-parametric Wilcoxon test to compare pairs of variables and each LCZ type to the median of research area. The Wilcoxon tests were also conducted in order to investigate if the LSTm differences in LCZs between past and present are significant. All the statistical calculations were conducted with the use of R programming language^53^, the spatial representations of the results were produced with QGIS (Hannover)^61^. Additional layers of the data (roads, water bodies and rivers) were obtained from Head Office of Geodesy and Cartography^66^.

## Results

### Relationship between the IMD and LST (MODIS)

**Figure 3:**
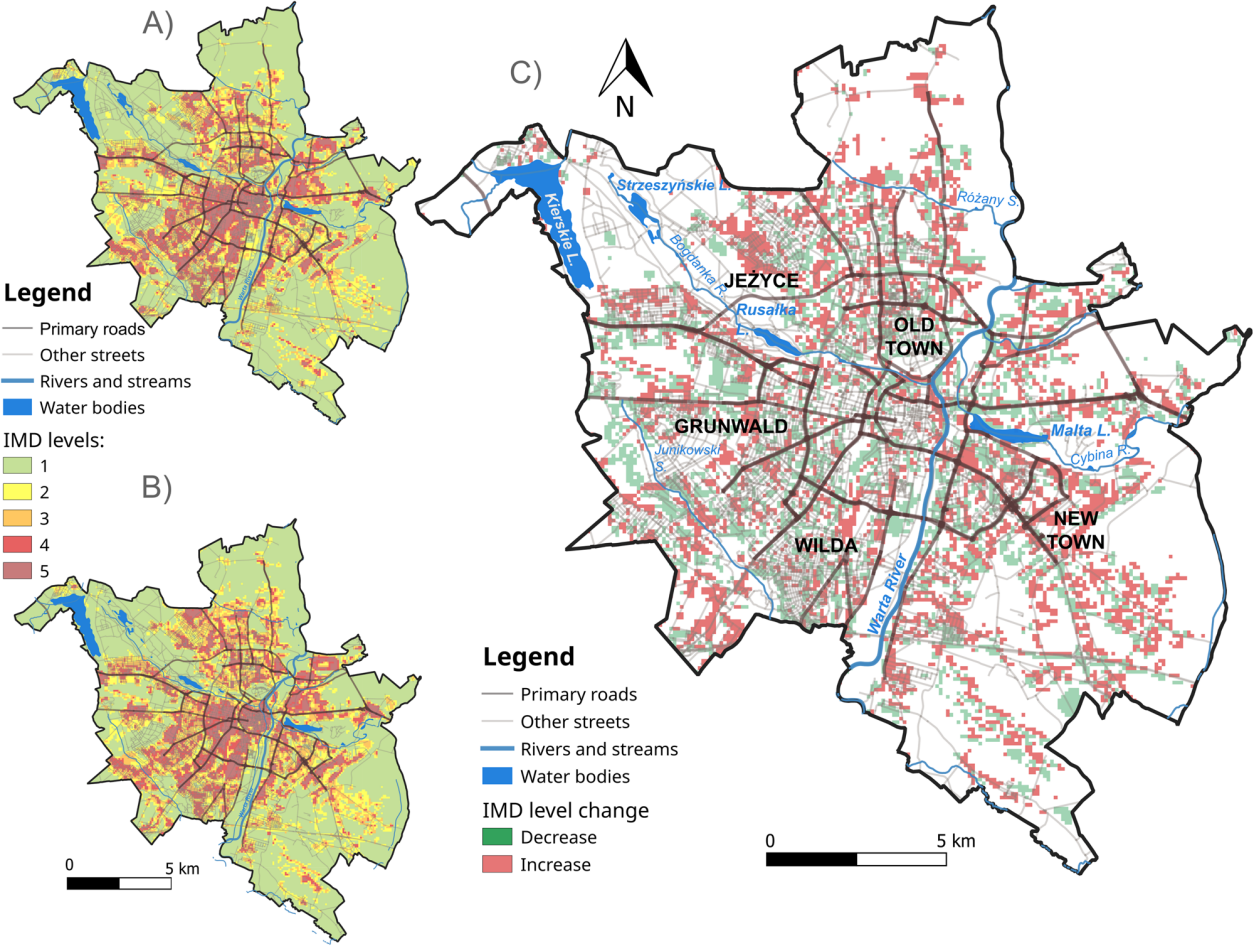
The spatial distribution of IMD levels in Poznań in 2006 (**A**), 2018 (**B**) and changes of IMD levels in Poznań between 2006 and 2018 (**C**) based on Copernicus’ imperviousness density (IMD) product.

Comparison of the IMD for 2006 and 2018 showed the highest increase of the areas of IMD levels 3 and 4, contrastingly the highest drop of IMD level 1. Regardless the change of IMD level, the average LST increased by ~ 1 °C (Table 2). The largest patches of areas where IMD levels increased are noted in the north and southeast of the city (Fig. 3).

**Table 2:** Changes of the areas (km^2^) covered by individual IMD levels between 2006 and 2018, and their LST change (°C) derived from MOD11A2v6 product for 2002–2011 and 2012–2021.

IMD level	IMD 2006 (km^2^)	IMD 2018 (km^2^)	Change of area (km^2^)	Change of MODIS average LST (°C)
1 (0–20%)	147.29	140.26	− 7.03	0.94
2 (21–40%)	36.85	36.93	0.08	0.97
3 (41–60%)	33.46	38.17	4.71	1.0
4 (61–80%)	27.4	29.12	1.72	1.05
5 (81–100%)	16.82	17.34	0.52	1.05

**Figure 4:**
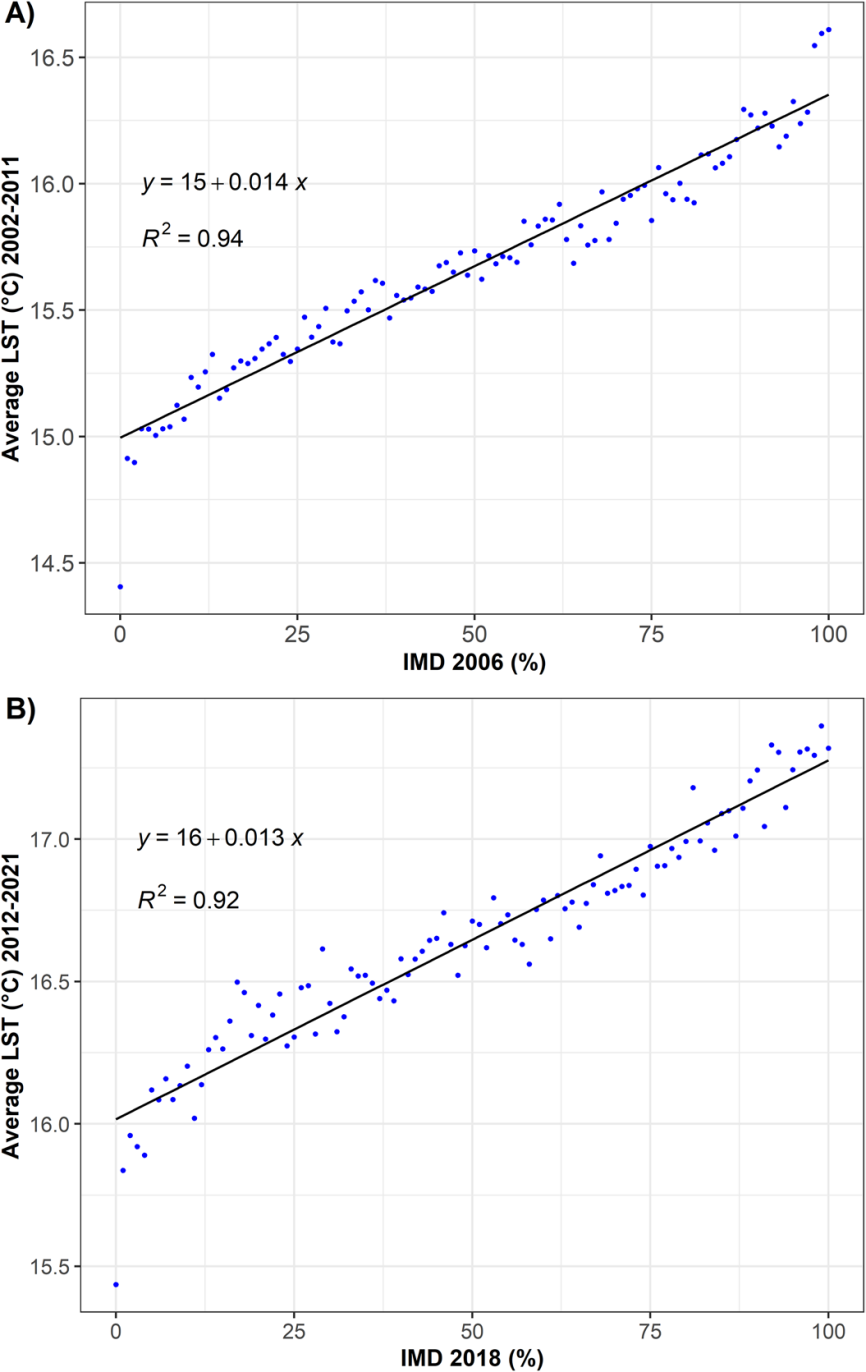
Linear regression models, the relationship between IMD and LST for: IMD 2006, 2002–2011 (**A**), and IMD 2018 and 2012–2021 (**B**).

The relationships between the LST and the IMD were found positive—on average, for every 10% increase in imperviousness, the surface temperature increases by up to 0.14 °C. Increase of the density of imperviousness of the area explains 92–94% of the observed LST’s rise (Fig. 4). Next, to investigate the influence of specific built-up classes on LST the LCZ classification was used.

### Map of local climate zones

**Figure 5:**
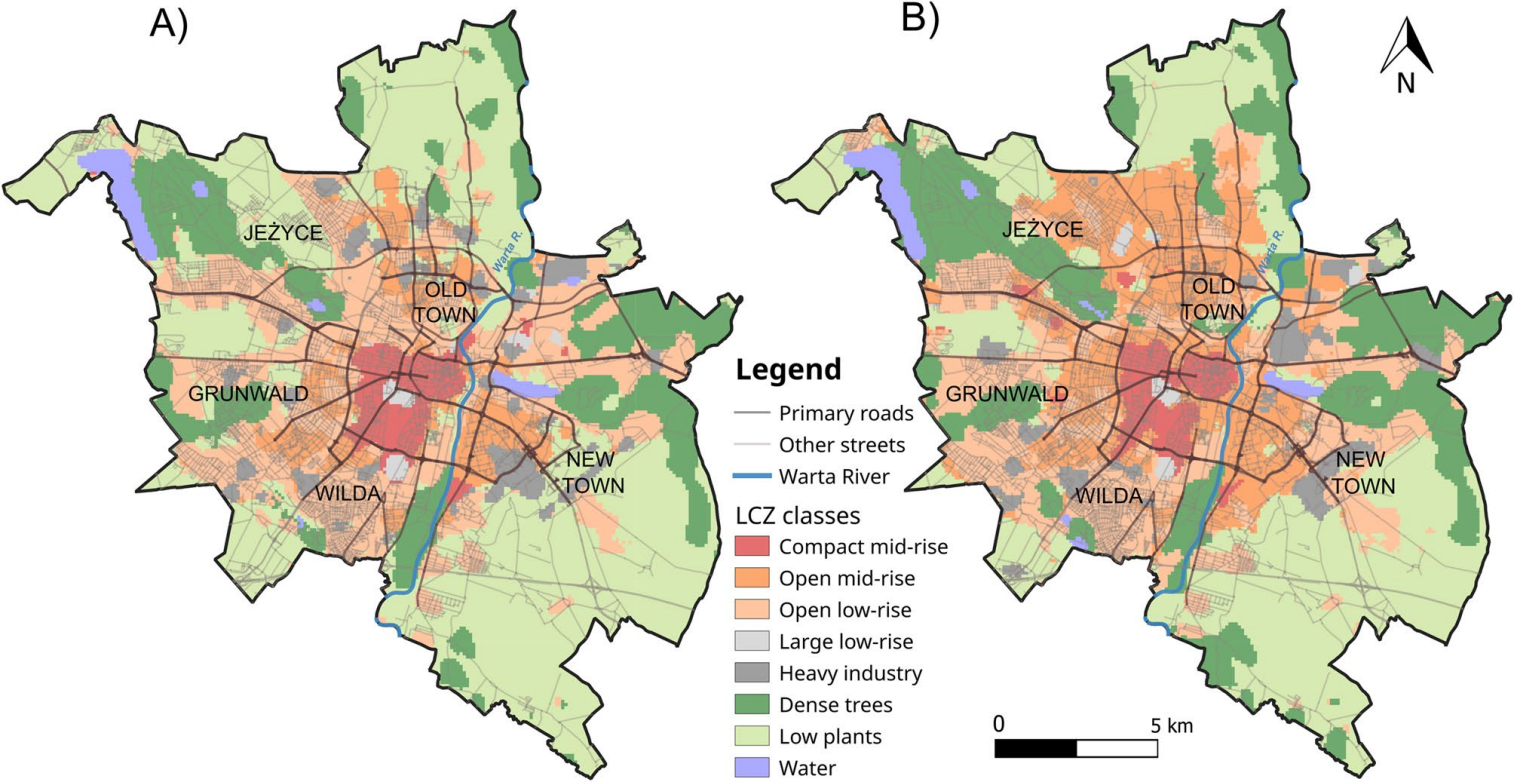
LCZ classes in Poznań; (**A**) 1988, (**B**) 2021.

The OA of LCZ maps was tested with the confusion matrix built using independent validation samples. The overall accuracy of LCZ maps are 89% and 87% for 1988 and 2021 respectively. The SAGA GIS classification maps shows progressing urbanisation in Poznań over the past 33 years (Fig. 5). The city developed its urban areas, especially on the northern, western and south-eastern parts. The urban areas increased mainly at the expense of low plants LCZ class.

**Figure 6:**
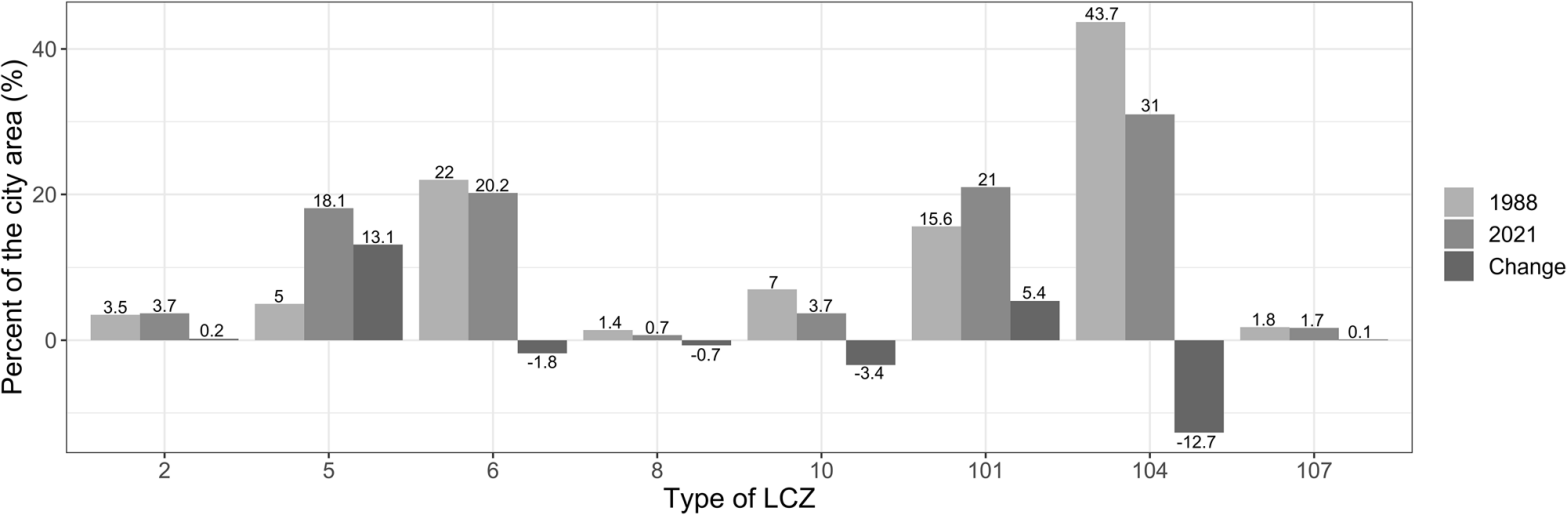
Changes in the area of LCZs in Poznań between 1988 and 2021 according to LCZ types (km^2^): Compact mid-rise [2], open mid-rise [5], open low-rise [6], large low-rise [8], heavy industry [10], dense trees [101], low plants [104], water [107].

Between 1988 and 2021 the overall contribution of urban areas increased from 102.1 km^2^ (38.9% of the city area) in 1988 to 121.31 km^2^ (46.4% of the city area) in 2021. The LCZ classes depending on land cover decreased from 159.8 km^2^ (61.1% of the city area) in 1988 to 140.50 km^2^ (53.7% of the city area) (Fig. 6, Table 3).

**Table 3:** Transformation of LCZs classes between 1988 and 2021 in Poznań (km^2^).

LCZ type		2021								
		Compact mid-rise	Open mid-rise	Open low-rise	Large low-rise	Heavy industry	Dense trees	Low plants	Water	Sum
1988	Compact mid-rise	7.54	1.08	0.12	0.06	0.03	0.36	0.01	0.05	9.25
	Open mid-rise	0.29	10.33	2.17	0.05	0.14	0.06	0.05	0	13.09
	Open low-rise	0.62	15.82	33.44	0	1.95	4.13	1.46	0.12	57.54
	Large low-rise	0.67	0.84	0.32	0.84	1.04	0	0.02	0	3.73
	Heavy industry	0.38	8.99	3.93	0.74	3.75	0.12	0.5	0	18.41
	Dense trees	0	0.11	0.91	0	0.02	39.36	0.21	0.12	40.73
	Low plants	0.06	10.06	12.05	0.06	2.65	10.42	78.82	0.22	114.34
	Water	0	0.18	0.03	0	0.05	0.46	0.06	3.95	4.73
	Sum	9.56	47.41	52.97	1.75	9.63	54.91	81.13	4.46	261.81

In the 1980s the areas classified by the model as heavy industry were mostly construction sites. The results presented in the Table 3 clearly illustrate it by showing the heavy industry LCZ classified areas of 1988 changed into open mid-rise and open low-rise LCZs in 2021. In the case of large low-rise LCZ class, changeability between 1988 and 2021 is caused by misclassifying it into heavy industry LCZ class and the fact that there were not many areas of this type existing in the past and remaining to the present day. Areas of low vegetation have largely changed into forest areas and transformed into urbanized areas, mainly open low-rise and open mid-rise, as well as heavy industry (Table 3).

**Figure 7:**
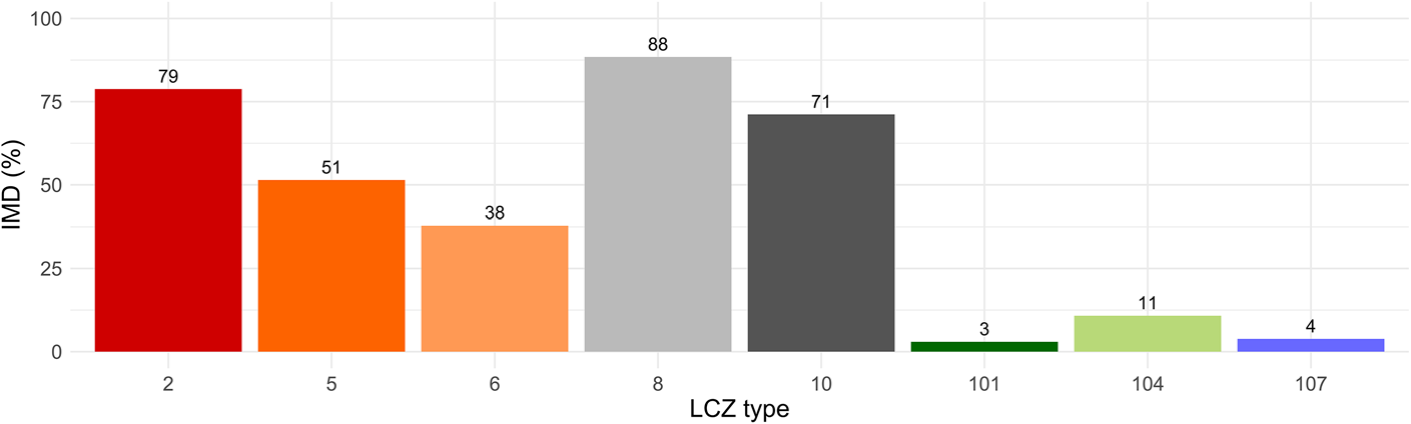
Estimated imperviousness density of LCZ classes in Poznań, based on IMD 2018 and LCZ 2021, Compact mid-rise [2], open mid-rise [5], open low-rise [6], large low-rise [8], heavy industry [10], dense trees [101], low plants [104], water [107] (colours of types according to Stewart & Oke^21^).

The large low-rise, compact mid-rise and heavy industry LCZs represent the highest average imperviousness density of the surface whereas built-up classes have medium IMD values (Fig. 7). It is factual that IMD of dense trees, low plants and water LCZs > 0 is related to the specific of LCZ classification^21^.

### Land surface temperature

Based on LST ~ IMD models and estimated imperviousness density of specific LCZs we can assume that the largest increase of LST occurs when dense trees, low plants and water LCZs transform into large low-rise, compact mid-rise and heavy industry LCZs (up to 1.19 °C in case of transformation from dense trees to large low-rise). Transformation of the aforementioned heavy built and compact classes into open mid-rise and open low-rise LCZ leads to the decrease of LST (up to − 0.70 °C in case of transformation from large low-rise to open low-rise). Moreover, change from open mid-rise to open low-rise also causes a decrease of LST. The transformation from any built-up class into semi-natural coverage leads to the drop of LST. However, the change from dense trees and water to low plants leads to its increase (Table 4).

**Table 4:** Estimated, predicted change in LST (°C) as a result of transformations between LCZ classes based on LST ~ IMD regression models.

LCZ type	Compact mid-rise	Open mid-rise	Open low-rise	Large low-rise	Heavy industry	Dense trees	Low plants	Water
Compact mid-rise		− 0.39	− 0.57	0.13	− 0.11	− 1.06	− 0.95	− 1.05
Open mid-rise	0.39		− 0.18	0.52	0.28	− 0.67	− 0.56	− 0.57
Open low-rise	0.57	0.18		0.70	0.46	− 0.49	− 0.38	− 0.48
Large low-rise	− 0.13	− 0.52	− 0.70		− 0.24	− 1.19	− 1.08	− 1.18
Heavy industry	0.11	− 0.28	− 0.46	0.24		− 0.95	− 0.84	− 0.94
Dense trees	1.06	0.67	0.49	1.19	0.95		0.11	0.01
Low plants	0.95	0.56	0.38	1.08	0.84	− 0.11		− 0.10
Water	1.05	0.57	0.48	1.18	0.94	0.01	0.10	

**Figure 8:**
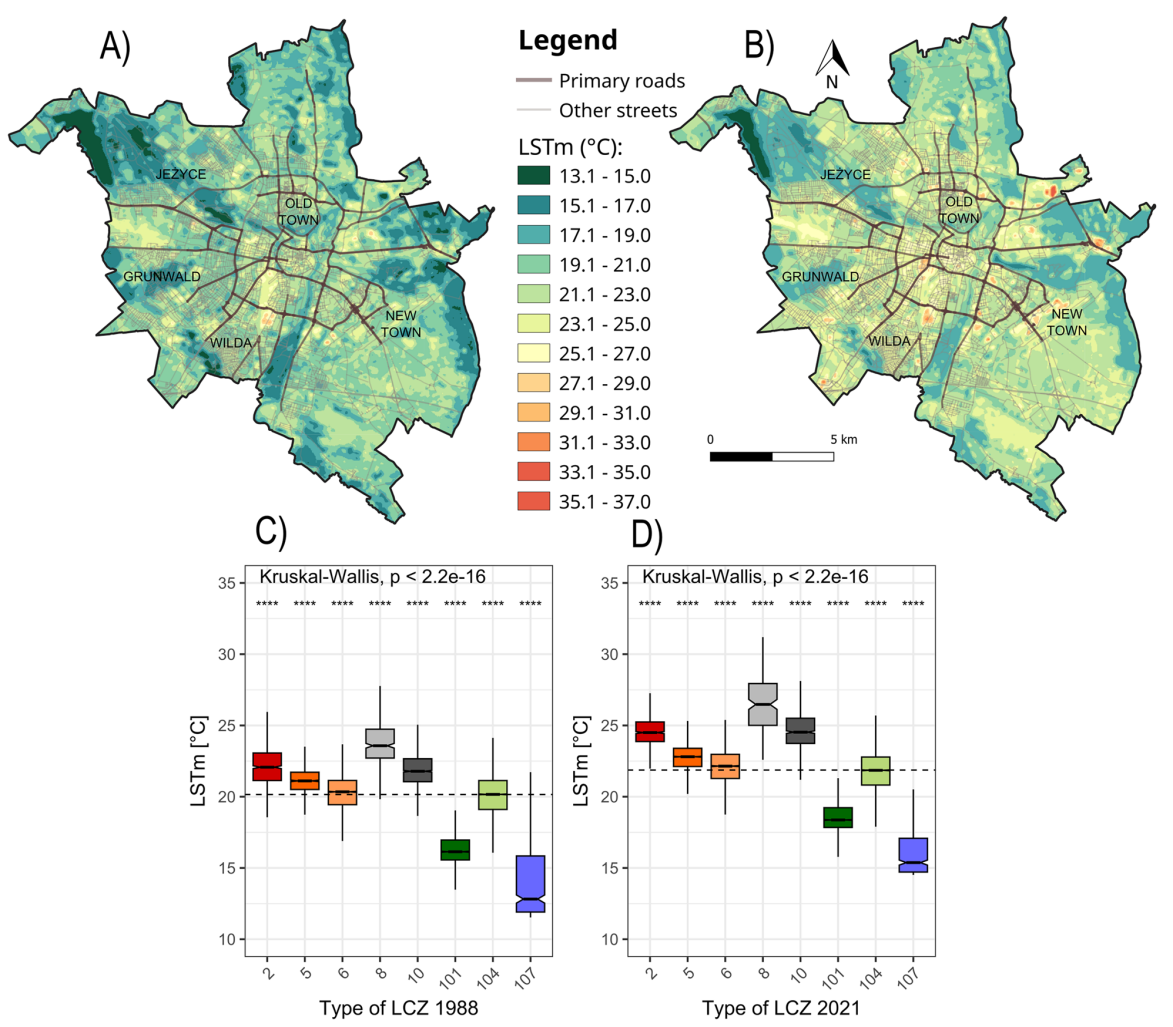
The mean LST (°C) in Poznań: (**A**) LSTm1 (1987–1989), (**B**) LSTm2 (2020–2022) based on Landsat 5 and 8 images and its statistics according to established LCZ units (**C**) LSTm1 ~ LCZ 1988 , (**D**) LSTm2 ~ LCZ 2021 (compact mid-rise [2], open mid-rise [5], open low-rise [6], large low-rise [8], heavy industry [10], dense trees [101], low plants [104], water [107]; colours of types according to Stewart & Oke^21^). The middle values denote the medians, the box extends to the Q1 (first quartile) and Q3 (third quartile), while the whiskers show a range of 99.3%: the upper whisker shows Q3 + 1.5*IQR (the interquartile range), the lower shows Q1 − 1.5*IQR. The notches extend to ± 1.58 IQR/sqrt(n) and show 95% confidence intervals. The dashed line indicates the median LSTm value for the research area. A pairwise comparison of Wilcoxon’s different test codes: ns—p > 0.05; *p ≤ 0.05; **p ≤ 0.01; ***p ≤ 0.001; ****p ≤ 0.000.

The highest LSTm values are noted around the city centre (compact mid-rise and large low-rise). The heavily increased LSTm is visible also within heavy industry LCZ. Lower LSTm occurs in open mid-rise and open low-rise LCZs. Dense trees and water LCZs cover the areas characterized by the lowest LSTm (Fig. 8). The average LSTm for the whole study area was 19.7 °C and 21.5 °C for 1988 and 2021 respectively.

The internal differences of the LST amid individual LCZ units in each period were tested. The differences of the LSTm of each LCZ class for each period were all found significant. There is an increase of the average LSTm between LSTm1 and LSTm2, with higher LSTm in LSTm2 (Fig. 8).

In both periods the higher mean, maximum and minimum LSTm are observed in LCZs units depending on land use. The characteristics of construction materials and the form of land use determine the increase of the LSTm compared to natural LCZs units based on land cover. From among the natural LCZs the low plants LCZ stands out with the highest LSTm comparable to open mid-rise and open low-rise LCZs. This correlation may be due to the fact that low plants are commonly situated between open housing areas.

Between 1988 and 2021 all the LSTm medians, minimums and maximums increased, besides with the maximum LSTm for water LCZ (Table 5). Differences of LSTm between specific LCZ classes of LSTm1 and LSTm2 were all found statistically significant with *p* ≤ 0.000. The most statistically relevant changes were found in the classes that underwent the broadest and most extensive transformations.

**Table 5:** Changes in the mean LST (LSTm) (°C) between periods 1987–1989 and 2020–2022 according to LCZ classes in Poznań.

LCZ type	Changes of LSTm			
	Mean LSTm	Median LStm	Maximum LSTm	Minimum LSTm
Compact mid-rise	2.61	2.43	2.67	7.39
Open mid-rise	1.68	1.69	4.14	2.24
Open low-rise	1.83	1.8	4.2	4.42
Large low-rise	2.97	2.94	6.01	2.76
Heavy industry	2.91	2.74	3.91	4.64
Dense trees	2.2	2.23	6.51	2.85
Low plants	1.75	1.69	2.25	3.81
Water	1.52	2.56	-0.46	2.98

The internal analysis on how transformation within specific classes affected LSTm showed the increase of the mean of LSTm even when certain classes remained unchanged. This finding suggests the possible impact of changes of global climate changes on LSTm. The results obtained have the tendencies corresponding with the created LST ~ IMD regression models (Fig. 4, Table 4). Transformation from seminatural coverage, low plants, into heavy industry led to the increase of the mean LSTm (1.75 °C). Change from heavy industry into open low-rise and open mid-rise LCZ classes led to the drop of the mean LSTm (up to − 1.20 °C) as well as transformation from open mid-rise to open low-rise (− 0.39 °C). Moreover, conversion from low plants LCZ to dense trees LCZ led to its decrease as well (− 0.62 °C) (Table 6).

**Table 6:** Change of LSTm (°C) (in relate to unchanged LCZ area) in specific LCZs depending on the direction of the transformation.

LCZ type	Compact mid-rise	Open mid-rise	Open low-rise	Large low-rise	Heavy industry	Dense trees	Low plants	Water
Compact mid-rise		− 0.27	− 0.12	1.00		− 0.26		
Open mid-rise	0.38		− 0.39		2.37	− 0.28		
Open low-rise	0.98	0.37			0.51	− 0.89	− 0.14	0.45
Large low-rise	− 0.90	− 1.26	− 1.15		− 0.73			
Heavy industry	0.01	− 0.63	− 1.20	1.77		− 0.57	− 0.90	
Dense trees		− 0.26	0.71				0.96	0.82
Low plants		0.25	− 0.24	2.40	1.75	− 0.62		0.32
Water		1.44				0.34		

Regression models rely on the relationship between LST and IMD. The subtraction of estimated, predicted change of LST of LSTm shows the potential size of the influence of factors other than IMD on LSTm (local or global). After excluding the estimated IMD impact on LSTm, it was found that LCZ transformations still led to the increase of the mean LSTm. The highest increase of LSTm due to LCZ conversion, excluding the impact of IMD, is noted in the case of conversion of open mid-rise LCZ into heavy industry LCZ (2.80 °C) and heavy industry LCZ to large low-rise LCZ (up to 2.52 °C) as well as low plants LCZ into large low-rise (1.48 °C). We may assume that the high values can partly be ascribed to the additional energy being released or accumulated in those heavy built areas (Table 7). These findings suggest the importance of factors, other than IMD, in shifts of the LSTm.

**Table 7:** Change of LSTm (°C) (in relate to unchanged LCZ area) excluding the estimated influence of IMD.

LCZ type	Compact mid-rise	Open mid-rise	Open low-rise	Large low-rise	Heavy industry	Dense trees	Low plants	Water
Compact mid-rise		0.45	0.41	1.98		− 0.21		
Open mid-rise	0.70		0.14		2.80	− 0.24		
Open low-rise	0.94	0.72			0.58	− 0.85	0.02	0.50
Large low-rise	0.21	− 0.55	− 0.62		0.26			
Heavy industry	0.89	0.09	− 0.66	2.52		− 0.53	− 0.74	
Dense trees		− 0.89	0.27				0.89	0.85
Low plants		− 0.16	− 0.47	1.48	1.06	− 0.57		0.38
Water		0.92				0.38		

## Discussion

Transformations of LULC affect the heat budget between the surface of the Earth and the atmosphere thereby modifying local climate^67^. The process of urbanisation is confirmed to be one of the main drivers of LST increase due to the intensive transformation of the environment’s spatial morphology. Moreover, it is projected to continue in the coming decades^12,68–70^. Therefore, in the first place we aimed to investigate this process on the research area for the past three decades.

Based on IMD data, it was found that in 12 years (2006–2018) the area of surfaces with imperviousness density of 41–100% increased by 6.95 km^2^ whereas those of 0–20% decreased by 7.03 km^2^. The application of the LCZ classification allowed the identification of the aforementioned areas as open mid-rise, compact mid-rise and heavy industry LCZs that IMD on the research area falls between 50 and 80%. The area of open mid-rise LCZ was found to be also increasing the most between 1988 and 2021. The development of residential housing areas is one of the main characteristics of the urbanisation processes directly linked with the population growth of cities^12^.

Generally, the contribution of built-up areas increased by over 7% (from 102.1 to 121.3 km^2^) between 1988 and 2021 even reaching 13% within open mid-rise LCZ. The open mid-rise class as well as other built-up LCZs increased at the cost of low plants, dense trees and open low-rise LCZs. Amid semi-natural LCZs, the areas of the low vegetation were intensively transformed. The intensive transformations of low plants LCZs due to urbanisation is commonly observed. For example Mushore et al.^28,29^ found this LCZ shrinking by 16.8% in 30 years while Hou et al.^71^ by 17.8% in only 12 years. In our research area during the 33-year period this LCZ shrunk by 13.1%. Moreover, Mushore et al.^28,29^ in their research on the period 2005–2020^29^ found the low plants LCZ increasing due to deforestation. In contrast, our research proved the decrease of low plants LCZ because of the reforestation. With high probability the noted increase of the dense trees areas is related to the transformations in Poland after 1989, when the green wedges of Poznań became again the concern of the authorities^72^.

Our results demonstrated that for every 10% increase in imperviousness, LST (MODIS) increases by up to 0.14 °C. The higher the IMD, the higher the LST which is in line with the results of previous studies^16,69^. Similar results were obtained by Cretu et al.^73^ in Iași (Romania) for the mean LST (2014–2018) for: winter night, autumn night, autumn day, summer night and spring night (0.16–0.21 °C) with the R^2^ 0.41–0.59. The authors obtained the highest values of R^2^ during summer day (0.78). Our calculations (R^2^ 0.92–0.94) on two separate decades and mean 8-day LST (MODIS) of all seasons have minimised the effect of the extremes and let us investigate the overall mean relationship of IMD and LST. We assumed that the largest increase of LST would occur due to transformation of seminatural LCZs into large low-rise LCZ (IMD > 90%). It is consistent with Mushore et al.^29^ who underlined that impervious surfaces and buildings and their high capacity of heat absorption causes elevation of LST.

Assumptions based on the models regarding the tendencies of the LST change due to certain LCZ transformations were in line with the LST obtained based on Landsat data. The conversion of seminatural areas into heavy built LCZ led up to 2.40 °C increase of the LSTm, while transformation of heavy industry into open built classes conversely led to its drop. The reforestation of the low plants LCZ led to − 0.62 °C decrease of LSTm. Additionally, according to the model and LSTm data, most of transformations into low plants LCZ have the lowest cooling effect of vegetated LCZ types. The tendencies are coherent with the findings on LST among certain LCZs described by Geletič et al.^74^ in two Central European cities (Prague and Brno), as well as the results of Nayak and Mandal^75^ on impact of LULC changes on LST in Western India and Zhou et al. in Guangzhou–Foshan, China^76^. We found the mean LSTm increasing in every LCZ between 1988 and 2021 even in areas where LCZ remained unchanged. That implies the warming is caused not only by LCZ transitions. Mushore et al.^28^ also registered the increase of LST within every LCZ class as well between 1990 and 2020. In accordance with the conclusions obtained by Mushore et al.^28,29^ we can suppose that the increase may be caused by the rise of the background temperature due to global warming.

LULC changes (according to both LCZ and IMD) significantly affect the LST—the relation between IMD and LST is positive. Moreover, considering only LCZs transformations, the LSTm in most LCZs is still rising. This finding suggests the importance of factors, other than IMD, in changes of LSTm, and are related to the specific properties of the LCZ classes^21^. For example, LSTm increase due to the transformations of seminatural areas into built-up ones, besides construction materials, is determined by the form of the land use—anthropogenic heat emissions. Moreover, the estimated drop in LSTm due to transformation from dense trees LCZ to open mid-rise LCZ may be caused due to the specifics of urban street canyons. The sky view factor as well as the modified airflow still affects the LSTm. Moreover, on urban areas tree canopies tend to be fragmented, which positively influences the LSTm^77^ possibly distorting the expected interclass dependencies. We may assume that the positive changes of LSTm as well as LST (MODIS), regardless of IMD level, are also connected with the rise of the background temperature^28,75^. Guo et al.^78^ in their research in Lagos estimated that global warming has a potential influence on the increase of urban LST reaching an average 39%. Moreover on our research area the increase of the annual temperatures related to the climate change is also noted in the in-situ measurements. In the period 1951–2000 the average annual temperature in Poznań was reaching 8.3 °C, with the highest in July (18.1 °C) and the lowest in January (− 1.6 °C)^79^. Meanwhile in the period 1991–2020 increased to 9.5 °C, 19.5 °C and -0.4 °C. Furthermore for the years 1848–2016 the air temperature in Poznań increased at a rate of 1.1 °C per 100 years. The air temperature increased the most in winter (+ 1.5 °C/100 years) and least in summer (+ 0.6 °C/100 years). Moreover, in the last three decades, the pace of the trend has increased to + 4.6 °C per 100 years, with the highest increase in summer (+ 7.5 °C/100 years)^43^. This has doubtless influenced the results of our research.

We conclude that the LULC changes have influence on LST. Moreover, we assume it is also affected by the change of the background temperature (global warming). This trend has been noted by other scientists^28,75,78^. Cities themselves have made a contribution to climate change due to their greenhouse gases emissions as well as the release of the additional heat and their high heat capacity. Therefore, besides focusing on apparent interventions aimed to decrease the LST induced by the LCZ changes, there is a need to focus also on other ways cities affect the global climate and to work on mitigating those impacts. The WUDAPT procedure involved to obtain the LCZ classification, allowed us to achieve the high accuracy results.

## Conclusions

The study investigated the impact of the 33-year period of changes of LULC on LST based on a medium-sized Central European city. The Landsat 5 and Landsat 8 data allowed to distinguish the LCZ classes on the research area using WUDAPT procedure with a satisfying accuracy (LCZ 1988 and LCZ 2021), as well as to estimate their LST characteristics (LSTm1 and LSTm2). The transformations of LCZ classes on the research area were registered along with their estimated effects on LST. The IMD and MODIS data utilised in the regression models allowed us to estimate the influence of impervious surfaces on LST, confirming their positive relationship and later to estimate the modelled LST changes due to transformations from one LCZ class to another. The tendencies of real and modelled LST changes due to LCZ transformations were found coinciding. The research confirmed that the transformations of LULC influenced LST but it was found that its changes may not only be impacted by local factors but also global ones.

The obtained results indicate the following:

The built-up areas in Poznań expanded. The seminatural LCZ that was fragmented the most due to the progressing urbanisation was low plants LCZ (shrunk by 33.21 km^2^). Among the urban areas the residential ones increased the most (up to 34 km^2^ in open mid-rise LCZ) indicating the active urbanisation processes.

For every 10% increase in IMD, LST increases by up to 0.14 °C. Based on our models the largest increase of LST (up to 1.19 °C) occurs due to the transformation of seminatural classes into the heavily built ones (compact mid-rise, large low-rise, heavy industry) characterised by the highest IMD.

The mean LST of every LCZ class increased comparing past and present times by up to 2.97 °C in the case of large low-rise LCZ with the lowest in water LCZ (1.52 °C).

The further internal analysis revealed that LST values increased even on areas with unchanged LCZs (on average by 1.9 °C). That suggests the impact of factors other than local. We can suppose that the increase may be caused by the rise of background temperature due to global warming.

The WUDAPT procedure allowed to receive overall relatively accurate results in our research but in the future better machine learning procedure for LCZ distinguishing of some of the classes (especially: large low-rise, heavy industry) would be a great improvement in the field. Moreover, further investigation on impacts of tree cover on local climate in specific LCZ classes on the research area would be demanded. Considering the directions of the changes noticed in the city and progressing climate change, the future investigations should be focused on the projections of the changes in LULC and LST, as well as the air temperature.

## Data Availability

The datasets generated during and/or analysed during the current study are available from the corresponding author on reasonable request.
